# Validating CRAVED: a brief screening instrument for ultra-processed food addiction

**DOI:** 10.3389/fpubh.2026.1871164

**Published:** 2026-08-13

**Authors:** Ellen Bennett, Hussein M Magdi, Fiachra McEnaney, Deborah Lycett, Jen Unwin

**Affiliations:** 1 Centre for Healthcare and Community Transformation, Coventry University, Coventry, United Kingdom; 2 Public Health Collaboration, London, United Kingdom; 3 The Collaborative Health Community Foundation, Oxford, United Kingdom; 4 North London NHS Foundation Trust, London, United Kingdom; 5 School of Biomedical Sciences, University of Leeds, Leeds, United Kingdom

**Keywords:** brief screening tool, CRAVED, food addiction, ICD-10, ultra-processed food, ultra-processed food use disorder, validation, Yale Food Addiction Scale

## Abstract

**Background:**

Addictive-like eating behaviours related to ultra-processed foods have gained increasing research and clinical attention. The Yale Food Addiction Scale (YFAS) is the most widely used measure of food addiction. It was primarily designed as research tool, so its length and scoring complexity may limit routine clinical use. CRAVED is a brief six-item screening tool developed to assess core addiction-related eating behaviours using a simple yes/no response format.

**Objective:**

This study aimed to provide a preliminary psychometric evaluation of CRAVED.

**Methods:**

A cross-sectional secondary analysis was conducted using anonymised baseline data from 117 adults enrolled in a community-based intervention for addictive-like eating behaviours. Participants completed CRAVED, YFAS 2.0, and the Binge Eating Scale (BES). Analyses examined internal consistency, construct validity, logistic regression, receiver operating characteristic (ROC) performance, and agreement with YFAS diagnostic classification.

**Results:**

CRAVED scores were positively associated with YFAS symptom scores (*r* = 0.55, *p* < 0.001) and BES scores (*r* = 0.56, *p* < 0.001). Higher CRAVED scores significantly predicted YFAS-defined food addiction. ROC analysis demonstrated acceptable discrimination (AUC = 0.75, 95% CI 0.64–0.85). A threshold of ≥3 showed high sensitivity for identifying YFAS-positive cases. CRAVED scores were significantly higher among participants meeting YFAS criteria and increased across binge eating severity groups. Internal consistency was modest (Cronbach’s *α* = 0.58, McDonald’s *ω* = 0.68), consistent with the brief multidomain binary structure of the tool.

**Conclusion:**

These findings provide preliminary support for CRAVED as a brief and clinically feasible screening tool for addictive-like eating behaviours. CRAVED may be useful for rapid identification in healthcare and community settings, although it should not be considered a standalone diagnostic instrument. Further validation in larger and more diverse samples is warranted.

## Introduction

There is an ongoing controversy over the existence of a substance use disorder relating to ultra-processed foods (1). Despite this, there exists a growing body of research alongside popular support for the condition to be recognized (2). Considerable research relating to the prevalence of food addiction (FA) symptoms has been undertaken using the Yale Food Addiction Scale (YFAS) (3). The scale was developed to follow the DSM criteria for substance use disorder (4) but in relation to processed food consumption. A recent meta-analysis of studies using YFAS has shown a 14% prevalence of food addiction in the general population and higher rates in clinical samples. For example, 55% of patients with binge eating disorder (BED) screened positive for food addiction on the YFAS (3).

Food addiction, as scored on the YFAS, is significantly related to the probability of suffering from type 2 diabetes (T2D) and mental health symptoms (5). Scoring positive for food addiction makes it 6.7 times more likely a person will have T2D and 2.4 times more likely they will have a mental health problem (6). Higher food addiction symptoms and the likelihood of T2D are related in a dose–response type manner (5). Findings such as these in the current climate of an epidemic of metabolic ill-health further underline the importance of advancing our understanding of addictive symptoms related to certain foods.

The YFAS was developed primarily for use in research settings (3) and is somewhat time consuming to score in the clinic. It has also been criticised for the overlap of some questions with binge eating symptoms (7).

The CRAVED tool (**C**ompulsion: powering urges to consume, **R**eaching: increase tolerance and need, **A**ctivities neglected: ignoring what you once valued, **V**olume: having more than you intended, **E**xclusion: causes withdrawal, **D**amage despite awareness, use continues) has been recently developed based on ICD criteria for substance use disorder (8, 9). The idea was to develop a tool which is easy to score in the clinic and which would overlap less with binge eating symptoms. CRAVED has a simple six question format with yes/no responses (8). A score of three or more indicates a possible addictive relationship with certain foods. CRAVED has been used in outcome research (9) and has been validated in Spanish. Seaman et al. (10) showed that Spanish YFAS and Spanish CRAVED had good concurrent validity, whereas CRAVED shared less variance with the Binge Eating Scale (BES) than YFAS. Spanish CRAVED thus seems to demonstrate strong agreement with Spanish YFAS and greater discrimination from Spanish BES (10).

This study is a preliminary validation of CRAVED in English. The overlap between CRAVED and already validated measures of food addiction (YFAS) and BED (BES) will be explored to ascertain the scale’s concurrent and discriminate validity with these measures.

CRAVED was expected to demonstrate acceptable internal consistency (*α* and *ω* ≥ 0.70) and a coherent underlying structure. Total CRAVED scores were expected to show moderate to strong positive correlations with YFAS scores, indicating convergent validity. Associations with BES were expected to be weaker than those with YFAS, supporting discriminant validity. Higher CRAVED scores were expected among participants meeting YFAS diagnostic criteria, and Receiver Operating Characteristics (ROC) analysis was expected to demonstrate discrimination above chance (AUC > 0.70).

## Method

### Study design

The study used a cross-sectional validation design based on existing, anonymised data. The analysis examined the psychometric properties of the CRAVED tool and compared it with two established measures: the Yale Food Addiction Scale (YFAS 2.0) (4) and the Binge Eating Scale (BES) (11). This study involved secondary analysis of data originally collected for programme evaluation (12). No additional data was collected for the purpose of this validation.

### Study setting

Data were collected as part of the Liberate programme feasibility and acceptability study (12), an online community-based intervention designed to support adults experiencing addictive-like eating or symptoms consistent with ultra-processed food addiction (UPFA). The current analysis used the Liberate programme dataset including two cohorts, a sample of 117 participants who completed baseline measures. Participants completed questionnaires remotely via a secure online platform.

### Participants

The dataset included 117 participants who completed all three questionnaires (CRAVED, YFAS 2.0, and BES). Participants had a mean age of approximately 50 years (SD = 12.72, range = 26–78) and were recruited through the Liberate programme, an online intervention for individuals experiencing symptoms consistent with ultra-processed food addiction (UPFA). Liberate is delivered under the umbrella of The Public Health Collaboration, a UK registered charity (Registration No. 1171887). Sociodemographic characteristics of the sample are available in Supplementary material.

The available sample size (*n* = 117) meets commonly cited recommendations for preliminary validation studies, which suggest a minimum of 100 participants and at least 5–10 participants per item. With six items in CRAVED, the sample was considered adequate to assess internal consistency and construct validity (10, 13).

### Sampling

A convenience sampling approach was used based on the existing dataset. No new participants were contacted, and no additional data was collected for this phase.

#### Inclusion criteria

- Adults (≥18 years)
- Completion of CRAVED, YFAS 2.0, and BES questionnaires

#### Exclusion criteria

- Missing responses for any of the key measures
- Incomplete consent or withdrawn consent

Demographic information (age, gender, BMI, and available health indicators) was used for descriptive statistics only. All data were collected under existing project ethical approval.

### Measures

#### CRAVED

CRAVED was developed by Unwin et al. (8) and consists of six self-report items assessing key features of addictive-like eating. Responses are recorded as yes/no. Total scores range from 0 to 6, with higher scores indicating greater addictive-like eating. A score of three or more indicates probable addiction based on ICD-10 substance-use criteria.

#### Yale Food Addiction Scale 2.0 (YFAS 2.0)

The YFAS 2.0 developed by Gearhardt et al. (4), is a validated questionnaire based on DSM substance-use criteria adapted to assess addictive-like eating behaviours. The scale includes 35 items and produces two summary scores: (a) a symptom count (0–11) and (b) a diagnostic classification with severity levels (mild, moderate, severe). Higher scores are associated with greater eating pathology and higher body weight.

#### Binge Eating Scale (BES)

The BES, developed by Gormally et al. (11), is a 16-item self-report measure assessing behavioural and emotional features of binge eating. Each item is scored from 0 to 3, producing a total score range of 0–46. Scores ≤17 indicate little or no binge eating, 18–26 indicate moderate severity, and ≥27 indicate severe binge eating.

#### Reporting guidelines

The study was reported in accordance with the STROBE statement for cross-sectional observational studies. Evaluation of measurement properties was informed by COSMIN recommendations for health-related measurement instruments.

#### Psychometric evaluation plan

The psychometric evaluation examined the reliability and validity of the CRAVED tool using established statistical methods. Internal consistency was assessed using Cronbach’s alpha and McDonald’s omega. Item–total correlations and “alpha if item deleted” statistics were used to evaluate item performance. Construct validity was assessed through convergent and discriminant analyses. Convergent validity was examined using associations between CRAVED and YFAS scores. Discriminant validity was evaluated by examining associations between CRAVED and BES scores. Additional evidence for validity included known-groups comparisons and diagnostic discrimination using ROC analysis.

### Statistical analysis

Analyses were conducted using SPSS (version 25) and R (version 4.3). Descriptive statistics summarised demographic characteristics and total scores. Item distributions were examined for floor and ceiling effects.

Internal consistency was evaluated using Cronbach’s alpha and McDonald’s omega to assess reliability of the CRAVED total score. Test–retest reliability could not be assessed due to the cross-sectional design. Structural validity could not be robustly examined due to binary responses, low item variability, and limited sampling adequacy. Assessment of factorability using tetrachoric correlations indicated inadequate sampling adequacy [Kaiser-Meyer-Olkin (KMO) = 0.48, Bartlett’s test = *p* < 0.001], suggesting insufficient shared variance among items to support robust factor analysis. The number of factors was determined using parallel analysis and inspection of the scree plot.

Construct validity was assessed using Spearman correlations. ROC analysis was conducted to evaluate the ability of CRAVED to distinguish between participants with and without YFAS-defined food addiction. Area Under the Curve (AUC), sensitivity, specificity, and optimal cut-off values were reported. Agreement between categorical classifications was examined using Cohen’s kappa (Supplementary material). Binary logistic regression analyses were conducted to examine whether CRAVED total scores and individual CRAVED items predicted YFAS diagnostic classification (coded 0 = no food addiction, 1 = food addiction). Odds ratios (ORs), 95% confidence intervals, and *p*-values were reported. These analyses were exploratory and intended to provide additional evidence regarding criterion-related validity.

Simple linear regression analyses were conducted to examine the extent to which CRAVED scores predicted YFAS symptom scores and BES total scores. Regression models were used as complementary analyses to quantify the proportion of variance explained (R^2^) beyond the correlation coefficients reported for construct validity. Spearman correlations were selected because several study variables deviated from normality assumptions. Linear regression models were subsequently conducted on summed scale scores to estimate explained variance and evaluate predictive associations. Diagnostic plots and model assumptions, including linearity, homoscedasticity, and residual distributions, were examined prior to interpretation.

### Missing data

A total of 121 participants were available in the original dataset. Participants with missing responses on any of the three primary measures (CRAVED, YFAS 2.0, or BES) were excluded from analysis. This resulted in a final complete-case sample of 117 participants. The proportion of excluded cases was 3.4%. Missing data were limited and therefore a complete-case approach was considered appropriate for this preliminary validation study.

## Results

Spearman correlations between CRAVED, YFAS, and BES scores are presented in Table 1. CRAVED demonstrated a moderate positive association with YFAS scores (*r* = 0.583, *p* < 0.001), supporting convergent validity against an established comparator measure (YFAS) of food addiction. A similar weight of association was observed between CRAVED and BES scores (*r* = 0.562, *p* < 0.001), demonstrating overlap with binge eating behaviours. However, as expected, YFAS and BES showed a stronger correlation (*r* = 0.741, *p* < 0.001) than CRAVED and BES, showing shared variance between food addiction and binge eating constructs.

**Table 1:** Spearman correlations between scales.

Variable	CRAVED	YFAS
YFAS	0.583 (0.000**)	1
BES	0.562 (0.000**)	0.741 (0.000**)

Sperman Correlation.

** Highly statistically significant at *p* ≤ 0.001.

Linear regression analyses were performed in Table 2 examining that CRAVED scores significantly predicted YFAS scores (*β* = 0.579, *p* < 0.001). This accounted for 33.5% of the variance, providing preliminary evidence of a moderate association between the constructs measured by CRAVED and YFAS. Similarly, CRAVED significantly predicted BES scores (*β* = 0.564, *p* < 0.001), explaining 31.8% of the variance, indicating a moderate relationship between CRAVED and binge-eating symptom severity. The relationship between YFAS and BES was stronger (*β* = 0.700, *p* < 0.001, *R*^2^ = 0.489), suggesting that these measures share a larger proportion of variance (see Table 2).

**Table 2:** Linear regression CRAVED and established measures.

Predictor	Outcome	*β*	*p*-value	R^2^
CRAVED	YFAS score	0.579	7.614 (0.000**)	0.335
CRAVED	BES score	0.564	7.327 (0.000**)	0.318
YFAS	BES	0.700	10.497 (0.000**)	0.489

* Statistically significant at *p* ≤ 0.05.

** Highly statistically significant at *p* ≤ 0.001.

Group comparisons of CRAVED scores are presented in Table 3, which shows participants who met YFAS diagnostic criteria reported significantly higher CRAVED scores (M = 5.28, SD = 0.97) compared to those who did not meet criteria (M = 4.10, SD = 1.42), with this difference reaching statistical significance (*p* < 0.001). CRAVED scores also showed a clear and statistically significant descent across binge eating severity categories. Participants with low BES scores reported the lowest CRAVED scores (M = 3.40, SD = 1.18), followed by those with mild–moderate binge eating (M = 4.90, SD = 1.10), and those with severe binge eating reporting the highest scores (M = 5.43, SD = 0.93) (*p* < 0.001).

**Table 3:** Group differences in CRAVED scores.

Group	CRAVED mean (SD)	*p*-value
YFAS diagnosis (No)	4.10 (1.42)	−5.023(0.000**)
YFAS diagnosis (Yes)	5.28 (0.97)	
BES low	3.40 (1.18)	24.206(0.000**)
BES mild–moderate	4.90 (1.10)	
BES sever	5.43 (0.93)	

** Highly statistically significant at *p* ≤ 0.001.

Item-level ROC analysis indicated variability in discriminatory performance across CRAVED items. CRAVED5 demonstrated the strongest discrimination (AUC = 0.78), with sensitivity of 0.87 and specificity of 0.70. CRAVED2 showed moderate discrimination (sensitivity = 0.86; specificity = 0.55), while CRAVED1 demonstrated high sensitivity (0.96) but low specificity (0.27). CRAVED6 similarly showed high sensitivity (0.93) but low specificity (0.22). Although CRAVED4 was included in the item-level ROC analyses, its near-universal endorsement (>99%) resulted in minimal variability and limited interpretability. Consequently, it was not emphasised in comparative interpretation of item performance (see Table 4).

**Table 4:** Receiver operating characteristic (ROC) analysis (CRAVED).

Item	AUC	CI_lower	CI_upper	Sensitivity	Specificity
CRAVED1	0.617	0.478	0.757	0.962	0.273
CRAVED2	0.707	0.618	0.796	0.861	0.553
CRAVED3	0.678	0.569	0.787	0.731	0.625
CRAVED4	0.514	0.487	0.540	1.000	0.027
CRAVED5	0.783	0.674	0.892	0.866	0.700
CRAVED6	0.576	0.474	0.678	0.929	0.222

**Figure 1:**
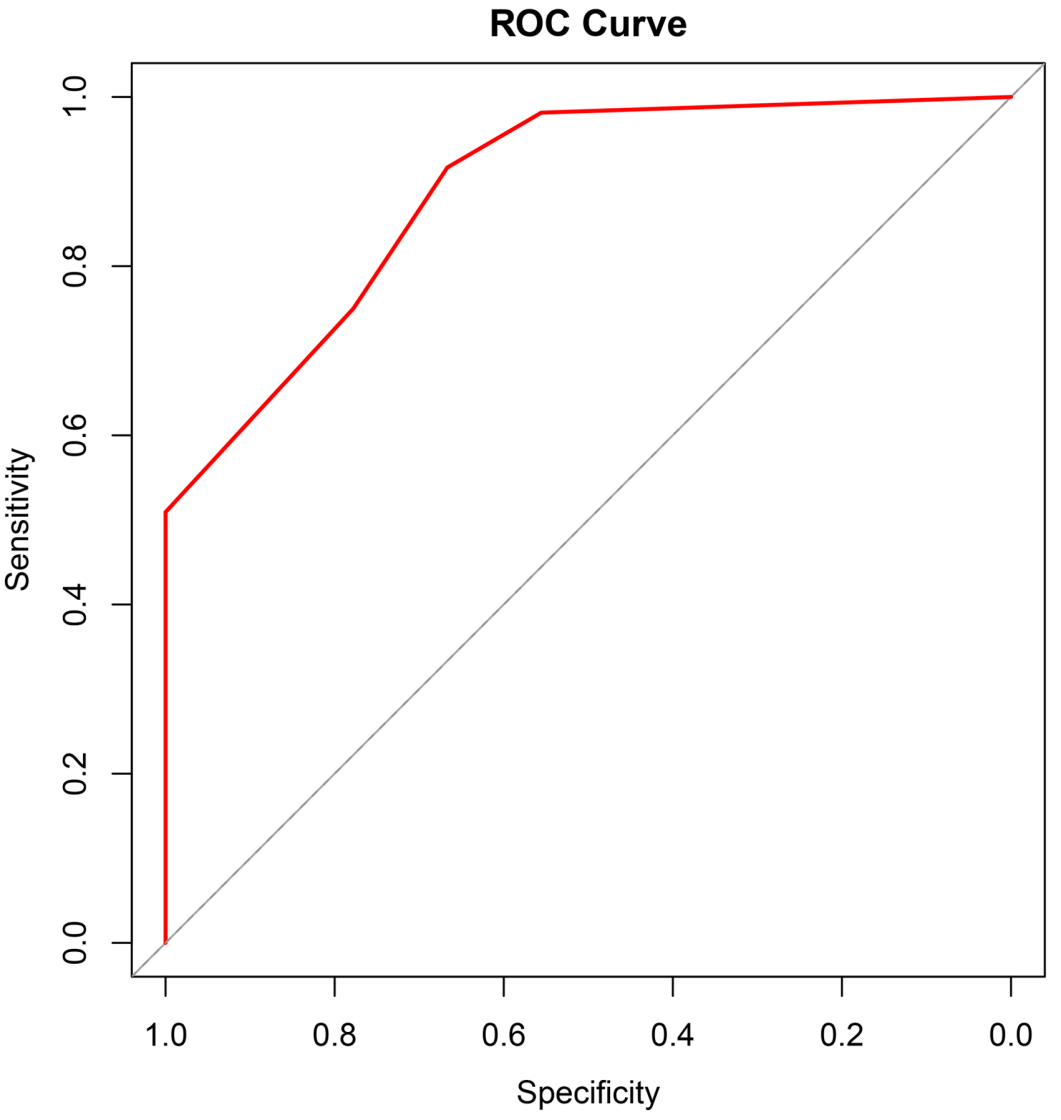
ROC analysis.

The ROC analysis demonstrated that the CRAVED total score showed acceptable discrimination in identifying YFAS-defined food addiction (AUC = 0.75, 95% CI [0.64–0.85], *p* < 0.001) (see Figure 1). Evaluation of selected cut-off points indicated that a threshold of ≥2 provided very high sensitivity (0.99) and specificity (0.79), while the predefined threshold of ≥3 maintained high sensitivity (0.94) with moderate specificity (0.66). A higher threshold of ≥4 resulted in reduced sensitivity (0.78) and specificity (0.48). Among the evaluated thresholds, a cut-off score of ≥2 produced the highest combined sensitivity and specificity (Youden Index = 0.782). However, because CRAVED was originally designed with a threshold of ≥3, both cut-points are presented and should be evaluated further in independent samples (see Tables 5, 6).

**Table 5:** Receiver operating characteristic (ROC) analysis (CRAVED) total score.

Item	AUC	SD Error	*p*-value	CI_lower	CI_upper
CRAVED	0.747	0.054	0.000**	0.641	0.852

** Highly statistically significant at *p* ≤ 0.001.

**Table 6:** Diagnostic performance of CRAVED at selected cut-off points.

Cut-off	Sensitivity	Specificity	Youden Index
≥2	0.989	0.793	0.782
≥3	0.943	0.655	0.598
≥4	0.784	0.483	0.267

Logistic regression analysis indicated that the CRAVED total score was a significant predictor of YFAS classification, with each one-point increase associated with substantially increased odds of meeting criteria (*β* = 1.35, OR = 3.85, 95% CI [2.10–8.52], *p* < 0.001) (see Table 7).

**Table 7:** Logistic regression (CRAVED).

Variable	Beta	OR	CI_lower	CI_upper	*p*_value
(Intercept)	−3.05	0.047	0.003	0.49	0.015*
CRAVED_total	1.347	3.845	2.096	8.524	0.000**

* Statistically significant at *p* ≤ 0.05.

** Highly statistically significant at *p* ≤ 0.001.

**Figure 2:**
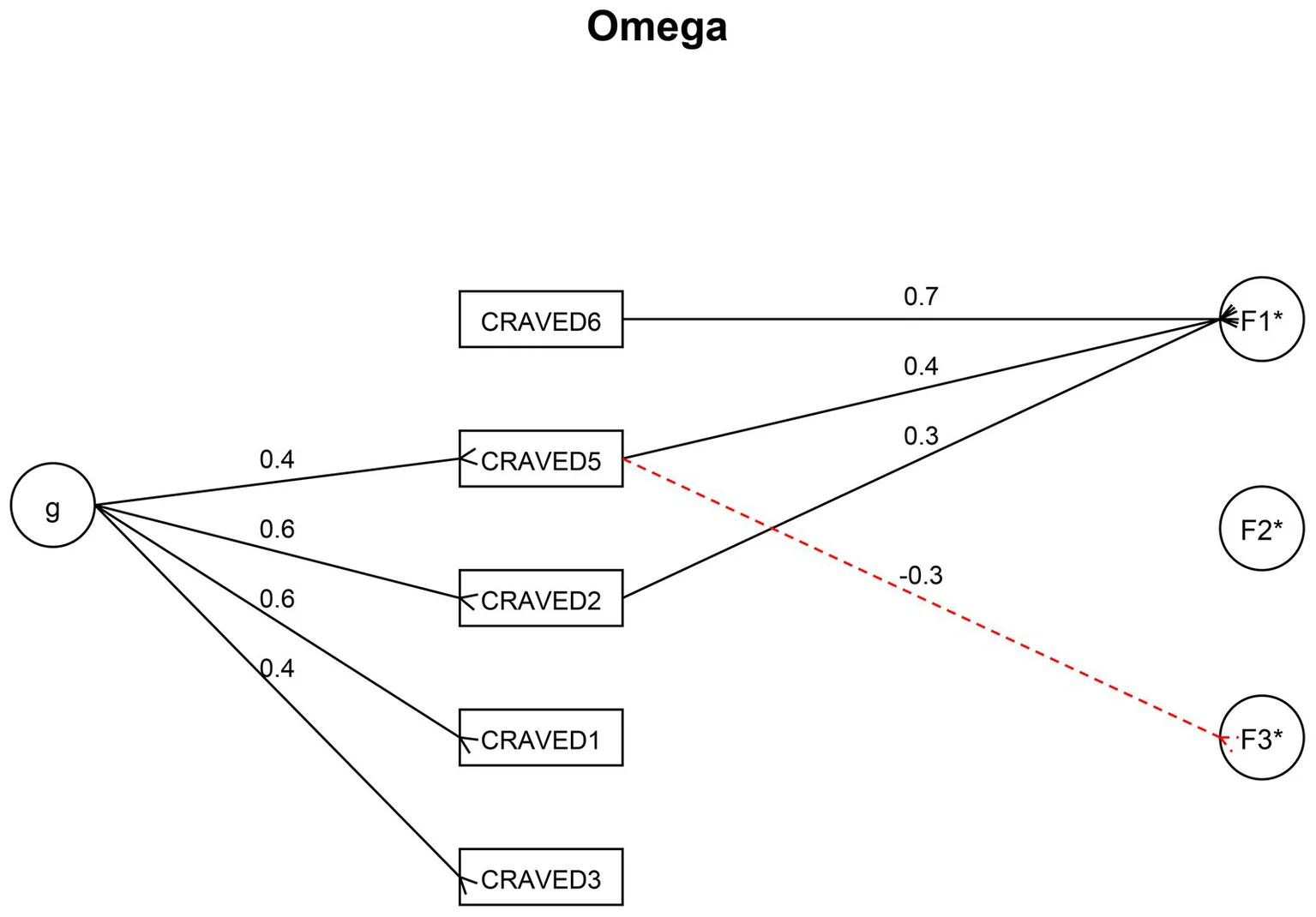
Schmid–Leiman factorial analysis of CRAVED items from McDonald’s omega.

Item-level logistic regression analyses demonstrated that most CRAVED items were significantly associated with YFAS classification. Endorsement of items was associated with increased odds of meeting criteria, with odds ratios ranging from 4.35 to 37.47. The strongest association was observed for CRAVED5 (OR = 37.47, 95% CI [6.35–716.71], *p* < 0.001), followed by CRAVED1 (OR = 13.00, 95% CI [2.18–74.06], *p* = 0.003) and CRAVED2 (OR = 11.62, 95% CI [2.62–81.43], *p* = 0.003). CRAVED6 (OR = 6.25, 95% CI [1.16–29.02], *p* = 0.022) and CRAVED3 (OR = 4.35, 95% CI [1.08–21.60], *p* = 0.046) showed comparatively smaller, but still significant, associations. The wide confidence intervals observed across several items suggest limited precision, reflecting high endorsement rates and sample size constraints (see Table 8). These findings should therefore be interpreted as exploratory. Standardised loadings are denoted by the general factor (g, addictive-like eating behaviour) and grouping factors (F1-F3, distinct item variability), which is presented in Figure 2. Correlation analysis indicated a significant positive association between CRAVED total score and YFAS symptom count (*r* = 0.55, 95% CI [0.41–0.67], *p* < 0.001), representing a moderate relationship (see Table 9).

**Table 8:** Logistic regression per item (CRAVED).

Item	OR	CI_lower	CI_upper	*p*_value
CRAVED1	13	2.175	74.056	0.003**
CRAVED2	11.62	2.616	81.428	0.003**
CRAVED3	4.353	1.082	21.604	0.046*
CRAVED5	37.474	6.353	716.711	0.001**
CRAVED6	6.25	1.156	29.017	0.022*

* Statistically significant at *p* ≤ 0.05.

** Highly statistically significant at *p* ≤ 0.001.

**Table 9:** Correlation analysis (CRAVED vs. YFAS).

Variable 1	Variable 2	*r*	CI_lower	CI_upper	*p*-value
CRAVED_total	YFAS_total	0.55	0.409	0.665	0.000**

** Highly statistically significant at *p* ≤ 0.001.

Exploratory and confirmatory factor analyses were not conducted. Preliminary assessment of factorability indicated inadequate sampling adequacy (Keyser-Meyer-Olkin (KMO) = 0.48), suggesting that the items did not share sufficient common variance to support a latent factor structure. Cronbach’s alpha (*α* = 0.6 CRAVED 1, 2, 3, 5 and 6) and McDonald’s omega total (*ω* = 0.68 CRAVED 1, 2, 3, 5 and 6) were moderate. McDonald’s ω hierarchical showed that ~48% of the total variance was explained by a single general factor, whereas McDonald’s ω asymptotic value showed that the upper limit of the reliability of CRAVED is ~70%. Inter-item reliability (ω = 0.24) showed that items were moderately related. Test–retest reliability was not available and should be examined in future studies. Internal consistency of the CRAVED items was moderate (Cronbach’s *α* = 0.58 CRAVED1-6), with inter-item correlations within an acceptable range (see Table 10).

**Table 10:** CRAVED item questionnaire Cronbach α consistency and McDonald’s ω reliability coefficients.

Measure	Value
Cronbach’s α	0.61
McDonald’s ω (Total)	0.68
McDonald’s ω (Hierarchial)	0.48
McDonald’s ω (Asymptotic)	0.70
Average inter-item correlation	0.24

## Discussion

This preliminary validation study showed that the CRAVED demonstrated moderate positive associations with both YFAS and BES scores, providing initial evidence of construct validity and supporting its potential utility as a brief screening measure of addictive-like eating behaviours. CRAVED captured core features of addictive-like eating. While it is not entirely distinct from related eating pathology (8, 9), YFAS showed more association with BES than CRAVED, suggesting that CRAVED may demonstrate less overlap with binge-eating symptoms than YFAS within the same sample. Linear regression between CRAVED and established measures revealed that the CRAVED tool has the capacity for overlapping behavioural and emotional components of eating pathology. These findings provide preliminary support for the construct validity of CRAVED as a measure of addictive-like eating, while also indicating that it captures related but not identical aspects of eating behaviour compared to YFAS and BES. Group difference comparisons between CRAVED, YFAS and BES indicated that CRAVED is sensitive to increasing severity of disordered eating behaviours and supports its utility as a brief screening tool for identifying clinically relevant eating pathology.

As CRAVED was developed as a composite screening tool, the primary focus of interpretation remains on total score performance rather than individual item analysis. Evaluation of ROC curves, internal consistency, and reliability indicates that CRAVED functions as a multi-item composite scale rather than a unidimensional measure. These results provide preliminary support for the existing cutoff score of ≥3 as a potentially useful screening threshold, although further validation in larger and more diverse samples is required. ROC analyses suggested that a threshold of ≥2 demonstrated the strongest overall discriminatory performance within the present sample. However, CRAVED was originally developed using a threshold of ≥3 based on conceptual and clinical considerations relating to endorsement of multiple addiction-related domains. For this reason, both thresholds are reported. Further research in larger and more diverse samples is required to determine the most appropriate operational cut-off for screening purposes. While individual items demonstrated varying predictive strengths, the total score appears to provide a useful summary indicator of addictive-like eating behaviours. Five of the six items significantly predicted binary (Yes/No) outcomes; however, CRAVED4 was excluded from item-level analysis due to near-universal endorsement (>99%; 116/117 participants), which resulted in unstable statistical estimates. Despite this low variability, CRAVED4 was retained within the total score because it captures a theoretically important component of the CRAVED framework. Given that the instrument contains only six items, removal of this item would substantially reduce content coverage and alter the balance of the conceptual domains represented. However, its near-universal endorsement within the present sample suggests limited discriminatory value in treatment-seeking populations with elevated symptom severity. This structure aligns with the design of CRAVED as a composite index of distinct behavioural domains rather than a psychometric tool reflecting a single underlying construct. Consequently, validation efforts prioritised criterion-related approaches, treating the scale as a multidimensional measure of unique but related eating behaviours.

The present findings suggest that CRAVED performs favourably in relation to established measures while serving a different clinical purpose. Unlike the Yale Food Addiction Scale 2.0, which was developed as a comprehensive research instrument based on DSM criteria and provides both symptom count and diagnostic severity, CRAVED was designed as a brief screening tool aligned with ICD substance-use concepts for the rapid identification of probable ultra-processed food addiction in routine practice (9). Its moderate association with YFAS scores provides preliminary support for convergent validity, indicating that CRAVED captures core features of addictive-like eating (4). Given its brevity and ease of scoring, CRAVED may be particularly useful for clinicians in time-limited settings. In the absence of other brief screening tools specifically developed for UPFA, CRAVED may help address an important clinical gap.

In contrast to the Binge Eating Scale, which focuses primarily on behavioural and emotional features of binge eating, CRAVED appears more specifically oriented toward addiction-related processes such as craving, impaired control, and continued use despite adverse consequences (14). The finding that CRAVED shared less variance with binge eating pathology than YFAS may indicate improved discrimination between overlapping but distinct conditions.

These findings are consistent with emerging international evidence, including validation of the Spanish version of CRAVED, which also reported good agreement with YFAS alongside clearer separation from binge eating measures (10). Taken together, the evidence suggests that CRAVED may offer a middle ground: briefer, more clinically feasible, and easier to score than YFAS, while more addiction-specific than binge-eating measures alone.

### Clinical implications of CRAVED

Recent research has highlighted the high prevalence of ultra-processed food addiction in healthcare settings (3) and its strong association with chronic conditions such as type 2 diabetes and mental health problems (5). Healthcare professionals could be aided with an appropriate validated screening tool for UPFA symptoms which is suitable for clinic use and can indicate where future management and treatment efforts may be best directed (10). The YFAS was developed as a research, rather than screening tool, data analysis and interpretation is time consuming and has some overlap with binge eating symptoms (4, 7, 12), making it less suitable for use in general healthcare. The current results suggest that CRAVED has potential as a quick and valid screening tool for UPFA symptoms which relates well to the current gold standard for UPFA symptoms (YFAS). CRAVED may be a useful guide for clinicians evaluating appropriate counselling and support efforts. As CRAVED is more distinct than YFAS from binge eating symptoms (2, 10), it may have potential to help clinicians distinguish the primary problem which is essential for effective intervention. The BES is also a brief screening tool and could be used alongside CRAVED in the clinic to distinguish or confirm UPFA and binge eating symptoms which can co-occur but also arise as separate disorders (2). The current findings are supported by previous data which found similar results in regard to the relationship between CRAVED, YFAS and the BES (10).

### Limitations, potential sources of Bias, and future directions

Several limitations should be considered when interpreting the current findings. First, the study utilised a convenient sample of participants enrolled in a community-based intervention and were therefore self-selected and treatment-seeking. This may limit generalisability to the wider population and may increase symptom severity and associations between measures (13). The high prevalence of food addiction within this sample likely influenced classification metrics, including specificity and agreement estimates. Second, all measures were self-reported, which may introduce response bias and shared method variance (15). Third, the relatively small sample size (*n* = 117) adequate for preliminary validation, may have contributed to instability in some estimates, particularly in item-level analyses where binary responses and low variability can produce unstable odds ratios and wide confidence intervals. Findings should therefore be considered preliminary (16). Although the Liberate programme collected data at multiple time points, the present analysis used baseline data only. The study was therefore cross-sectional and did not permit assessment of test–retest reliability, responsiveness, or predictive validity.

Additionally, the CRAVED tool demonstrated limited variability in one item (CRAVED4), which may warrant further refinement in future iterations. The absence of a clear latent factor structure suggests that CRAVED functions as a composite index rather than a unidimensional scale, which has implications for how reliability and validity should be conceptualised. Future research should aim to validate CRAVED in larger and more diverse populations, including non-treatment-seeking samples, to better assess its generalisability and specificity. Findings may generalise primarily to treatment-seeking adults with elevated addictive-like eating symptoms. Longitudinal studies would also be valuable in evaluating test–retest reliability, responsiveness to change, and predictive validity. Further work may explore refinement of individual items and optimisation of cut-off thresholds to enhance diagnostic performance.

## Conclusion

This study provides preliminary evidence supporting the validity and clinical utility of the CRAVED tool as a brief screening measure for addictive-like eating behaviours. CRAVED demonstrated acceptable discriminatory ability, strong sensitivity, and meaningful associations with established measures of food addiction and binge eating. These findings suggest that CRAVED captures core features of addictive-like eating in a manner consistent with existing constructs, while offering a simpler and more accessible format for use in applied settings.

Importantly, the high sensitivity observed supports the role of CRAVED as an effective screening tool, particularly in clinical and community contexts where rapid identification is required. However, lower specificity and modest agreement with YFAS indicate that CRAVED should not be used as a standalone diagnostic instrument. Instead, it is best positioned as an initial step in assessment, with positive cases requiring further evaluation.

Future research should aim to validate CRAVED in larger and more diverse populations, refine item performance, and explore longitudinal properties including responsiveness to change and predictive validity. With further development, CRAVED has the potential to contribute meaningfully to the identification and management of addictive-like eating behaviours within public health and clinical practice.

## Data Availability

The data analyzed in this study is subject to the following licenses/restrictions: GDPR. Requests to access these datasets should be directed to Ellen.Bennett@phcuk.org.

## References

[1] Lustig RH. The battle over "food addiction". Front Psych. (2025) 16:1621742. doi: 10.3389/fpsyt.2025.1621742, 40950761 PMC12423058

[2] Unwin J Giaever H Avena N Kennedy C Painschab M Lafata EM. Toward consensus: using the Delphi method to form an international expert consensus statement on ultra-processed food addiction. Front Psych. (2025b) 16:1542905. doi: 10.3389/fpsyt.2025.1542905, 40375880 PMC12078235

[3] Praxedes DRS Silva-Junior AE Macena ML Oliveira AD Cardoso KS Nunes LO . Prevalence of food addiction determined by the Yale food addiction scale and associated factors: a systematic review with meta-analysis. Eur Eat Disord Rev. (2022) 30:85–95. doi: 10.1002/erv.2878, 34953001

[4] Gearhardt AN Corbin WR Brownell KD. Development of the Yale food addiction scale version 2.0. Psychol Addict Behav. (2016) 30:113–21. doi: 10.1037/adb0000136, 26866783

[5] Horsager C Bruun JM Faerk E Hagstrom S Lauritsen MB Ostergaard SD. Food addiction is strongly associated with type 2 diabetes. Clin Nutr. (2023) 42:717–21. doi: 10.1016/j.clnu.2023.03.014, 36996685

[6] Ozdemir EK Icen S Doger E Torun YT Solmaz N Camurdan MO . The relationship between food addiction, eating attitudes, and psychiatric symptoms with metabolic control in adolescents with type 1 diabetes mellitus. J Eat Disord. (2025) 13:58. doi: 10.1186/s40337-025-01242-w, 40176195 PMC11966810

[7] Graver H Mccausland HC Kruger M Rubino LG Moussaoui JR Parnarouskis L . Unique and shared symptoms across food addiction and binge-eating measures: a content analysis. Int J Eat Disord. (2026) 59:885–95. doi: 10.1002/eat.70033, 41550070

[8] Unwin J Delon C Giaever H Kennedy C Painschab M Sandin F . Low carbohydrate and psychoeducational programs show promise for the treatment of ultra-processed food addiction. Front Psych. (2022) 13:1005523. doi: 10.3389/fpsyt.2022.1005523, 36245868 PMC9554504

[9] Unwin J Delon C Giaever H Kennedy C Painschab M Sandin F . Low carbohydrate and psychoeducational programs show promise for the treatment of ultra-processed food addiction: 12-month follow-up. Front Psych. (2025a) 16:1556988. doi: 10.3389/fpsyt.2025.1556988, 40357512 PMC12067479

[10] Seaman J Apodaca P Morales JA Unwin J Giaever H Soto-Mota A. Statistical evidence of psychometric differences between bing eating disorder and food addiction screening tools among Spanish speaking patients with non-communicable metabolic diseases. Research Square. (2025). doi: 10.21203/rs.3.rs-6877570/v1

[11] Gormally J Black S Daston S Rardin D. The assessment of binge eating severity among obese persons. Addict Behav. (1982) 7:47–55. doi: 10.1016/0306-4603(82)90024-7, 7080884

[12] Bennett E Lycett D Whelan M Bellamy EL Banks S Patel R. A feasibility and acceptability study of liberate: an online, peer-supported, psychoeducational intervention for ultra processed food addiction. Front Psych. (2025) 16:1620372. doi: 10.3389/fpsyt.2025.1620372, 41234427 PMC12605181

[13] Terwee CB Bot SD De Boer MR Van Der Windt DA Knol DL Dekker J . Quality criteria were proposed for measurement properties of health status questionnaires. J Clin Epidemiol. (2007) 60:34–42. doi: 10.1016/j.jclinepi.2006.03.012 17161752

[14] Kalan RE Smith A Mason TB Smith KE. Independent associations of food addiction and binge eating measures with real-time eating behaviors and contextual factors: an exploratory ecological momentary assessment study. Appetite. (2024) 192:107127. doi: 10.1016/j.appet.2023.107127, 37980955 PMC10843748

[15] Podsakoff PM Mackenzie SB Lee JY Podsakoff NP. Common method biases in behavioral research: a critical review of the literature and recommended remedies. J Appl Psychol. (2003) 88:879–903. doi: 10.1037/0021-9010.88.5.879, 14516251

[16] Devellis RF. Scale Development: Theory and Applications. Thousand Oaks: Sage (2017).

