# Supplementary material for: Validating CRAVED: a brief screening instrument for ultra-processed food addiction
Source: Front Public Health. 2026 Aug 13;14:1871164. doi: 10.3389/fpubh.2026.1871164 (PMC13518580; doi:10.3389/fpubh.2026.1871164)
Supplement: Supplementary file 1 [file Table_1.docx]

# CRAVED Supplementary

### Table Conceptual Mapping of YFAS (DSM-5) and CRAVED (ICD-10) Criteria

| Core Addiction Domain | DSM-5 Substance Use Criteria (YFAS basis) | YFAS Operationalisation | ICD-10 Dependence Criteria (CRAVED basis) | CRAVED Item |
| --- | --- | --- | --- | --- |
| Loss of control | Substance taken in larger amounts or longer than intended | Eating more than intended; unsuccessful attempts to cut down | Difficulties controlling use (onset, termination, levels) | Volume – consuming more than intended |
| Craving / compulsion | Craving or strong desire to use substance | Persistent desire or urges to consume certain foods | Strong desire or compulsion to take the substance | Compulsion – powerful urges to consume |
| Tolerance (escalation) | Need for increased amounts to achieve effect | Increasing consumption over time to achieve satisfaction | Evidence of tolerance (increased amounts required) | Reaching – increased tolerance and need |
| Withdrawal | Withdrawal symptoms or use to relieve withdrawal | Distress or negative states when not consuming certain foods | Physiological withdrawal state when use is reduced or stopped | Exclusion – withdrawal symptoms when not consuming |
| Time/priority shift | Great deal of time spent obtaining/using/recovering | Time spent thinking about or engaging in food-related behaviours | Progressive neglect of alternative pleasures or interests | Activities neglected – neglect of previously valued activities |
| Continued use despite harm | Continued use despite physical or psychological problems | Continued consumption despite awareness of harm | Persistent use despite clear evidence of harmful consequences | Damage – continued use despite awareness of harm |
| Functional impairment | Failure to fulfil major role obligations | Impact on daily functioning (work, social roles) | Implied within harmful use and dependence pattern | Reflected across multiple CRAVED items |
| Diagnostic threshold | ≥2 symptoms (mild), ≥4 (moderate), ≥6 (severe) | Symptom count + impairment/distress criteria | ≥3 criteria for dependence diagnosis | ≥3 “yes” responses indicates probable addiction |

### Table Item-Level Mapping of YFAS and CRAVED Criteria

| Addiction Domain | CRAVED Item | CRAVED Description | YFAS Items |
| --- | --- | --- | --- |
| Loss of control / compulsion | **C – Compulsion** | Powerful urges to consume | 4, 25, 31, 32, 29, 30 |
| Tolerance (escalation) | **R – Reaching for more** | Increased tolerance and need | 24, 26 |
| Time / priority shift | **A – Activities neglected** | Ignoring what you once valued | 5, 6, 7, 8, 10, 18, 20 |
| Loss of control (behavioural) | **V – Volume** | Having more than intended | 1, 2, 3 |
| Withdrawal | **E – Exclusion** | Elimination causes withdrawal | 11, 12, 13, 14, 15 |
| Continued use despite harm | **D – Damage** | Use continues despite awareness | 22, 23, 28, 33, 34 |
| Functional impairment / distress | (implicit across CRAVED) | Not directly assessed as a separate item | 9, 21, 35, 19, 27, 16, 17 |
| Diagnostic threshold | ≥3 items endorsed | Binary (yes/no) | ≥2 symptoms + impairment/distress |

### Table YFAS vs CRAVED Items Threshold.

| Criteria | YFAS | CRAVED |
| --- | --- | --- |
| Once a month | 9, 10, 19, 27, 33, 35 | Binary (yes/no) |
| Two or three times a month | 8, 18, 20, 21, 34 |  |
| Once a week | 3, 11 ,13 ,14, 22, 28, 29 |  |
| Two or three times a week | 5, 12, 16, 17, 23, 24, 26, 30, 31, 32 |  |
| Four to six times a week | 1, 2, 4, 6, 7, 15, 25 |  |

### Table CRAVED Items.

Question 1: Have you ever had such a strong desire or sense of compulsion at the thought of having these foods or drinks that you could not resist the urge to consume them?

Question 2: Have you ever noticed that you need to use increasing amounts of these foods or drinks to get the same effect compared to when you first had them?

Question 3: Have you ever noticed that you neglect planning activities because you are too tired, sick or preoccupied due to having too much of these foods or drinks?

Question 4: Have you ever consumed more of these foods or drinks than you intended on more than one occasion?

Question 5: Have you ever experienced at least two of the following withdrawal symptoms when you cut down or stopped consuming these foods or drinks? (Multiple selection)

- Headache

- Nausea or vomiting

- Anxiety

- Depressed or low mood

- Irritability

- Shakes

- Sweating

- Heart racing / palpitations

- Fast or shallow breathing

- Diarrhoea or constipation

- Sleep disturbance or vivid dreams

- None of the above

- Other

Question 6: Have you ever continued to consume these foods or drinks despite you or someone else believing that difficulties with weight gain, diabetes, memory, concentration, anxiety, unexplained mood swings, depression, panic attacks or other physical or mental health problems could be due to your consumption of these foods or drinks?

### Table Frequencies of CRAVED Clinical Assessment Tool. (N = 117)

|  | Yes (%) | No (%) | Mean (SD) | Mean Rank | Median | Mode |
| --- | --- | --- | --- | --- | --- | --- |
| CRAVED1 | 110 (94.02) | 7 (5.98) | 0.94 (0.24) | 2 | 1 | 1 |
| CRAVED2 | 85 (72.65) | 32 (27.35) | 0.73 (0.45) | 5 | 1 | 1 |
| CRAVED3 | 77 (65.81) | 40 (34.19) | 0.66 (0.48) | 6 | 1 | 1 |
| CRAVED4 | 116 (99.15) | 1 (0.85) | 0.99 (0.09) | 1 | 1 | 1 |
| CRAVED5 | 90 (76.92) | 27 (23.08) | 0.77 (0.42) | 4 | 1 | 1 |
| CRAVED6 | 106 (90.60) | 11 (9.40) | 0.91 (0.29) | 3 | 1 | 1 |

SD: Standard Deviation

### Table Frequencies of YFAS. (N = 117)

| ITEM | 0 (%) | 1 (%) | 2 (%) | 3 (%) | 4 (%) | 5 (%) | 6 (%) | 7 (%) | MEAN (SD) | Mean  RANK | Median | Mode |
| --- | --- | --- | --- | --- | --- | --- | --- | --- | --- | --- | --- | --- |
| YFAS01 | 0 (0.0) | 1 (0.9) | 3 (2.6) | 4 (3.4) | 10 (8.5) | 36 (30.8) | 27 (23.1) | 36 (30.8) | 5.58 (1.31) | 3 | 6 | 5 |
| YFAS02 | 0 (0.0) | 1 (0.9) | 1 (0.9) | 9 (7.7) | 9 (7.7) | 37 (31.6) | 32 (27.4) | 28 (23.9) | 5.46 (1.28) | 4 | 6 | 5 |
| YFAS03 | 19 (16.2) | 21 (17.9) | 11 (9.4) | 13 (11.1) | 20 (17.1) | 18 (15.4) | 11 (9.4) | 4 (3.4) | 2.96 (2.12) | 24 | 3 | 1 |
| YFAS04 | 0 (0.0) | 2 (1.7) | 3 (2.6) | 7 (6.0) | 6 (5.1) | 30 (25.6) | 20 (17.1) | 49 (41.9) | 5.69 (1.48) | 2 | 6 | 7 |
| YFAS05 | 0 (0.0) | 9 (7.7) | 3 (2.6) | 13 (11.1) | 16 (13.7) | 28 (23.9) | 21 (17.9) | 27 (23.1) | 4.90 (1.78) | 8 | 5 | 5 |
| YFAS06 | 3 (2.6) | 7 (6.0) | 5 (4.3) | 8 (6.8) | 17 (14.5) | 37 (31.6) | 20 (17.1) | 20 (17.1) | 4.74 (1.79) | 11 | 5 | 5 |
| YFAS07 | 3 (2.6) | 12 (10.3) | 10 (8.5) | 9 (7.7) | 24 (20.5) | 32 (27.4) | 16 (13.7) | 11 (9.4) | 4.17 (1.85) | 17 | 5 | 5 |
| YFAS08 | 31 (26.5) | 25 (21.4) | 9 (7.7) | 8 (6.8) | 12 (10.3) | 19 (16.2) | 9 (7.7) | 4 (3.4) | 2.50 (2.26) | 27 | 2 | 0 |
| YFAS09 | 55 (47.0) | 21 (17.9) | 7 (6.0) | 11 (9.4) | 9 (7.7) | 6 (5.1) | 2 (1.7) | 6 (5.1) | 1.61 (2.09) | 29 | 1 | 0 |
| YFAS10 | 55 (47.0) | 19 (16.2) | 12 (10.3) | 13 (11.1) | 5 (4.3) | 7 (6.0) | 1 (0.9) | 5 (4.3) | 1.52 (1.97) | 30 | 1 | 0 |
| YFAS11 | 10 (8.5) | 12 (10.3) | 8 (6.8) | 20 (17.1) | 13 (11.1) | 26 (22.2) | 11 (9.4) | 17 (14.5) | 3.89 (2.16) | 21 | 4 | 5 |
| YFAS12 | 16 (13.7) | 15 (12.8) | 5 (4.3) | 12 (10.3) | 18 (15.4) | 16 (13.7) | 15 (12.8) | 20 (17.1) | 3.79 (2.41) | 22 | 4 | 7 |
| YFAS13 | 8 (6.8) | 7 (6.0) | 6 (5.1) | 14 (12.0) | 10 (8.5) | 26 (22.2) | 15 (12.8) | 31 (26.5) | 4.60 (2.18) | 14 | 5 | 7 |
| YFAS14 | 20 (17.1) | 19 (16.2) | 16 (13.7) | 16 (13.7) | 6 (5.1) | 16 (13.7) | 8 (6.8) | 16 (13.7) | 3.10 (2.40) | 23 | 3 | 0 |
| YFAS15 | 1 (0.9) | 5 (4.3) | 6 (5.1) | 9 (7.7) | 10 (8.5) | 18 (15.4) | 20 (17.1) | 48 (41.0) | 5.38 (1.86) | 5 | 6 | 7 |
| YFAS16 | 2 (1.7) | 4 (3.4) | 5 (4.3) | 11 (9.4) | 12 (10.3) | 26 (22.2) | 19 (16.2) | 38 (32.5) | 5.17 (1.82) | 6 | 5 | 7 |
| YFAS17 | 13 (11.1) | 16 (13.7) | 17 (14.5) | 5 (4.3) | 10 (8.5) | 13 (11.1) | 12 (10.3) | 31 (26.5) | 3.92 (2.57) | 20 | 4 | 7 |
| YFAS18 | 29 (24.8) | 23 (19.7) | 15 (12.8) | 12 (10.3) | 14 (12.0) | 9 (7.7) | 8 (6.8) | 7 (6.0) | 2.45 (2.21) | 28 | 2 | 0 |
| YFAS19 | 36 (30.8) | 22 (18.8) | 6 (5.1) | 11 (9.4) | 9 (7.7) | 14 (12.0) | 5 (4.3) | 14 (12.0) | 2.57 (2.51) | 25 | 2 | 0 |
| YFAS20 | 56 (47.9) | 20 (17.1) | 13 (11.1) | 12 (10.3) | 5 (4.3) | 6 (5.1) | 2 (1.7) | 3 (2.6) | 1.41 (1.85) | 32 | 1 | 0 |
| YFAS21 | 61 (52.1) | 22 (18.8) | 10 (8.5) | 9 (7.7) | 7 (6.0) | 2 (1.7) | 4 (3.4) | 2 (1.7) | 1.24 (1.78) | 33 | 0 | 0 |
| YFAS22 | 7 (6.0) | 7 (6.0) | 10 (8.5) | 7 (6.0) | 9 (7.7) | 21 (17.9) | 11 (9.4) | 45 (38.5) | 4.87 (2.28) | 10 | 5 | 7 |
| YFAS23 | 4 (3.4) | 9 (7.7) | 8 (6.8) | 12 (10.3) | 7 (6.0) | 19 (16.2) | 11 (9.4) | 47 (40.2) | 4.95 (2.22) | 7 | 5 | 7 |
| YFAS24 | 11 (9.4) | 11 (9.4) | 5 (4.3) | 14 (12.0) | 10 (8.5) | 24 (20.5) | 15 (12.8) | 27 (23.1) | 4.29 (2.32) | 16 | 5 | 7 |
| YFAS25 | 0 (0.0) | 1 (0.9) | 5 (4.3) | 5 (4.3) | 9 (7.7) | 26 (22.2) | 21 (17.9) | 50 (42.7) | 5.71 (1.47) | 1 | 6 | 7 |
| YFAS26 | 14 (12.0) | 14 (12.0) | 8 (6.8) | 11 (9.4) | 7 (6.0) | 23 (19.7) | 14 (12.0) | 26 (22.2) | 4.03 (2.47) | 19 | 5 | 7 |
| YFAS27 | 63 (53.8) | 18 (15.4) | 4 (3.4) | 7 (6.0) | 5 (4.3) | 13 (11.1) | 4 (3.4) | 3 (2.6) | 1.51 (2.13) | 31 | 0 | 0 |
| YFAS28 | 27 (23.1) | 2 (1.7) | 5 (4.3) | 9 (7.7) | 9 (7.7) | 21 (17.9) | 15 (12.8) | 29 (24.8) | 4.04 (2.66) | 18 | 5 | 7 |
| YFAS29 | 8 (6.8) | 11 (9.4) | 2 (1.7) | 13 (11.1) | 14 (12.0) | 22 (18.8) | 16 (13.7) | 31 (26.5) | 4.56 (2.23) | 15 | 5 | 7 |
| YFAS30 | 3 (2.6) | 7 (6.0) | 8 (6.8) | 16 (13.7) | 13 (11.1) | 23 (19.7) | 18 (15.4) | 29 (24.8) | 4.69 (1.99) | 13 | 5 | 7 |
| YFAS31 | 2 (1.7) | 3 (2.6) | 8 (6.8) | 14 (12.0) | 12 (10.3) | 34 (29.1) | 16 (13.7) | 28 (23.9) | 4.88 (1.77) | 9 | 5 | 5 |
| YFAS32 | 1 (0.9) | 6 (5.1) | 12 (10.3) | 14 (12.0) | 9 (7.7) | 34 (29.1) | 14 (12.0) | 27 (23.1) | 4.71 (1.87) | 12 | 5 | 5 |
| YFAS33 | 84 (71.8) | 16 (13.7) | 6 (5.1) | 0 (0.0) | 2 (1.7) | 5 (4.3) | 1 (0.9) | 3 (2.6) | 0.75 (1.64) | 35 | 0 | 0 |
| YFAS34 | 81 (69.2) | 18 (15.4) | 4 (3.4) | 3 (2.6) | 4 (3.4) | 3 (2.6) | 1 (0.9) | 3 (2.6) | 0.79 (1.63) | 34 | 0 | 0 |
| YFAS35 | 39 (33.3) | 18 (15.4) | 14 (12.0) | 9 (7.7) | 7 (6.0) | 6 (5.1) | 5 (4.3) | 19 (16.2) | 2.51 (2.61) | 26 | 2 | 0 |

0 = Never; 1 = Less than monthly; 2 = Once a month; 3 = 2–3 times a month; 4 = Once a week; 5 = 2–3 times a week; 6 = 4–6 times a week; 7 = Every day
SD: Standard Deviation

### Table Frequencies of Criteria Met vs Not Met for YFAS Individual Items. (N = 117)

| ITEM | Criteria Met (%) | Criteria Not Met (%) | Cut-off Frequency Required  to Meet Threshold | Numerical Cut-off (≥) |
| --- | --- | --- | --- | --- |
| YFAS01 | 54 (46.15) | 63 (53.85) | Four to six times a week | 6 |
| YFAS02 | 57 (48.72) | 60 (51.28) | Four to six times a week | 6 |
| YFAS03 | 64 (54.70) | 53 (45.30) | Once a week | 4 |
| YFAS04 | 48 (41.03) | 69 (58.97) | Four to six times a week | 6 |
| YFAS05 | 41 (35.04) | 76 (64.96) | Two to three times a week | 5 |
| YFAS06 | 77 (65.81) | 40 (34.19) | Four to six times a week | 6 |
| YFAS07 | 90 (76.92) | 27 (23.08) | Four to six times a week | 6 |
| YFAS08 | 65 (55.56) | 52 (44.44) | Two to three times a month | 3 |
| YFAS09 | 76 (64.96) | 41 (35.04) | Once a month | 2 |
| YFAS10 | 74 (63.25) | 43 (36.75) | Once a month | 2 |
| YFAS11 | 50 (42.74) | 67 (57.26) | Once a week | 4 |
| YFAS12 | 66 (56.41) | 51 (43.59) | Two to three times a week | 5 |
| YFAS13 | 35 (29.91) | 82 (70.09) | Once a week | 4 |
| YFAS14 | 71 (60.68) | 46 (39.32) | Once a week | 4 |
| YFAS15 | 49 (41.88) | 68 (58.12) | Four to six times a week | 6 |
| YFAS16 | 34 (29.06) | 83 (70.94) | Two to three times a week | 5 |
| YFAS17 | 61 (52.14) | 56 (47.86) | Two to three times a week | 5 |
| YFAS18 | 67 (57.26) | 50 (42.74) | Two to three times a month | 3 |
| YFAS19 | 58 (49.57) | 59 (50.43) | Once a month | 2 |
| YFAS20 | 89 (76.07) | 28 (23.93) | Two to three times a month | 3 |
| YFAS21 | 93 (79.49) | 24 (20.51) | Two to three times a month | 3 |
| YFAS22 | 31 (26.50) | 86 (73.50) | Once a week | 4 |
| YFAS23 | 40 (34.19) | 77 (65.81) | Two to three times a week | 5 |
| YFAS24 | 51 (43.59) | 66 (56.41) | Two to three times a week | 5 |
| YFAS25 | 46 (39.32) | 71 (60.68) | Four to six times a week | 6 |
| YFAS26 | 54 (46.15) | 63 (53.85) | Two to three times a week | 5 |
| YFAS27 | 81 (69.23) | 36 (30.77) | Once a month | 2 |
| YFAS28 | 43 (36.75) | 74 (63.25) | Once a week | 4 |
| YFAS29 | 34 (29.06) | 83 (70.94) | Once a week | 4 |
| YFAS30 | 47 (40.17) | 70 (59.83) | Two to three times a week | 5 |
| YFAS31 | 39 (33.33) | 78 (66.67) | Two to three times a week | 5 |
| YFAS32 | 42 (35.90) | 75 (64.10) | Two to three times a week | 5 |
| YFAS33 | 100 (85.47) | 17 (14.53) | Once a month | 2 |
| YFAS34 | 103 (88.03) | 14 (11.97) | Two to three times a month | 3 |
| YFAS35 | 57 (48.72) | 60 (51.28) | Once a month | 2 |

### Table Frequencies of BES. (N = 117)

| Item | 0 | 1 | 2 | 3 | Mean (SD) | Mean Rank | Median | Mode |
| --- | --- | --- | --- | --- | --- | --- | --- | --- |
| BES01 | 4 (3.42) | 8 (6.84) | 74 (63.25) | 31 (26.50) | 2.13 (0.68) | 2 | 2 | 2 |
| BES02 | 27 (23.08) | 18 (15.38) | 44 (37.61) | 28 (23.93) | 1.62 (1.09) | 10 | 2 | 2 |
| BES03 | 5 (4.27) | 53 (45.30) | 33 (28.21) | 26 (22.22) | 1.68 (0.87) | 9 | 2 | 1 |
| BES04 | 2 (1.71) | 11 (9.40) | 55 (47.01) | 49 (41.88) | 2.29 (0.71) | 1 | 2 | 2 |
| BES05 | 3 (2.56) | 28 (23.93) | 75 (64.10) | 11 (9.40) | 1.80 (0.63) | 5 | 2 | 2 |
| BES06 | 2 (1.71) | 47 (40.17) | 68 (58.12) | - | 1.56 (0.53) | 12 | 2 | 2 |
| BES07 | 7 (5.98) | 36 (30.77) | 49 (41.88) | 25 (21.37) | 1.79 (0.85) | 6 | 2 | 2 |
| BES08 | 16 (13.68) | 23 (19.66) | 57 (48.72) | 21 (17.95) | 1.71 (0.92) | 8 | 2 | 2 |
| BES09 | 20 (17.09) | 35 (29.91) | 47 (40.17) | 15 (12.82) | 1.49 (0.92) | 14 | 2 | 2 |
| BES10 | 4 (3.42) | 33 (28.21) | 65 (55.56) | 15 (12.82) | 1.78 (0.71) | 7 | 2 | 2 |
| BES11 | 11 (9.40) | 61 (52.14) | 40 (34.19) | 5 (4.27) | 1.33 (0.71) | 15 | 1 | 1 |
| BES12 | 19 (16.24) | 44 (37.61) | 18 (15.38) | 36 (30.77) | 1.61 (1.09) | 11 | 1 | 1 |
| BES13 | 16 (13.68) | 45 (38.46) | 35 (29.91) | 21 (17.95) | 1.52 (0.94) | 13 | 1 | 1 |
| BES14 | 2 (1.71) | 39 (33.33) | 38 (32.48) | 38 (32.48) | 1.96 (0.85) | 4 | 2 | 1 |
| BES15 | 0 (0.00) | 36 (30.77) | 49 (41.88) | 32 (27.35) | 1.97 (0.76) | 3 | 2 | 2 |
| BES16 | 14 (11.97) | 74 (63.25) | 29 (24.79) | - | 1.13 (0.60) | 16 | 1 | 1 |

SD: Standard Deviation

### Table Item-Level Comparison (CRAVED vs YFAS)

| CRAVED Item | Domain | CRAVED Description | YFAS Items (Underlying DSM Criterion) | YFAS Composite Variable |
| --- | --- | --- | --- | --- |
| C – Compulsion | Craving / urge | Powerful urges to consume | 4, 25, 29, 30, 31, 32 | YFAS_CRAVING |
| R – Reaching | Tolerance | Increased tolerance and need | 24, 26 | YFAS_TOLERANCE |
| A – Activities neglected | Time / priority shift | Ignoring valued activities | 5, 6, 7, 8, 10, 18, 20 | YFAS_TIME |
| V – Volume | Loss of control | Eating more than intended | 1, 2, 3 | YFAS_LOSS_CONTROL |
| E – Exclusion | Withdrawal | Symptoms when stopping | 11, 12, 13, 14, 15 | YFAS_WITHDRAWAL |
| D – Damage | Continued use despite harm | Use despite awareness | 22, 23, 28, 33, 34 | YFAS_DAMAGE |
|  | Impairment | Functional distress | 9, 16, 17, 19, 21, 27, 35 | YFAS_IMPAIRMENT |

### Table Item-Level Correlation between CRAVED and YFAS.

| CRAVED | YFAS | r (p – value) | Correlation |
| --- | --- | --- | --- |
| CRAVED1 | B-YFAS4 | 0.290 (0.001**) | Weak Positive |
|  | B-YFAS25 | 0.220 (0.017*) | Weak Positive |
|  | B-YFAS29 | 0.420 (0.000**) | Moderate Positive |
|  | B-YFAS30 | 0.379 (0.000**) | Weak Positive |
|  | B-YFAS31 | 0.289 (0.002**) | Weak Positive |
|  | B-YFAS32 | 0.386 (0.000**) | Weak Positive |
| CRAVED2 | B-YFAS24 | 0.433 (0.000**) | Moderate Positive |
|  | B-YFAS26 | 0.477 (0.000**) | Moderate Positive |
| CRAEVD3 | B-YFAS5 | 0.335 (0.000**) | Weak Positive |
|  | B-YFAS6 | 0.216 (0.019*) | Weak Positive |
|  | B-YFAS7 | 0.292 (0.001**) | Weak Positive |
|  | B-YFAS8 | 0.504 (0.000**) | Moderate Positive |
|  | B-YFAS10 | 0.311 (0.001**) | Weak Positive |
|  | B-YFAS18 | 0.508 (0.000**) | Moderate Positive |
|  | B-YFAS20 | 0.347 (0.000**) | Weak Positive |
| CRAVED4 | B-YFAS1 | 0.041 (0.659) | Weak Positive |
|  | B-YFAS2 | 0.180 (0.052) | Weak Positive |
|  | B-YFAS3 | 0.086 (0.356) | Weak Positive |
| CRAVED5 | B-YFAS11 | 0.547 (0.000**) | Moderate Positive |
|  | B-YFAS12 | 0.560 (0.000**) | Moderate Positive |
|  | B-YFAS13 | 0.487 (0.000**) | Moderate Positive |
|  | B-YFAS14 | 0.473 (0.000**) | Moderate Positive |
|  | B-YFAS15 | 0.476 (0.000**) | Moderate Positive |
| CRAVED6 | B-YFAS22 | 0.137 (0.142) | Weak Positive |
|  | B-YFAS23 | 0.311 (0.001**) | Weak Positive |
|  | B-YFAS28 | 0.138 (0.138) | Weak Positive |
|  | B-YFAS33 | 0.112 (0.229) | Weak Positive |
|  | B-YFAS34 | 0.158 (0.090) | Weak Positive |

r Spearman Correlation
* Statistically significant at p ≤ 0.05
** Highly statistically significant at p ≤ 0.001.

### Table Participant Characteristics (N = 117)

| Variable | Category | n | % | Mean (SD) | Min–Max |
| --- | --- | --- | --- | --- | --- |
| Age Range | 25–34 | 17 | 14.53 | 50.09 | 26 – 78 |
|  | 35–44 | 24 | 20.51 | (12.72) |  |
|  | 45–54 | 31 | 26.50 |  |  |
|  | 55–64 | 28 | 23.93 |  |  |
|  | 65–74 | 14 | 11.97 |  |  |
|  | 75–84 | 3 | 2.56 |  |  |
| Sex | Male | 14 | 11.97 |  |  |
|  | Female | 103 | 88.03 |  |  |
| Ethnicity | Asian or Asian British – Bangladeshi | 1 | 0.85 |  |  |
|  | Asian or Asian British – Indian | 4 | 3.42 |  |  |
|  | Asian or Asian British – Pakistani | 1 | 0.85 |  |  |
|  | Asian or Asian British – Any other Asian background | 1 | 0.85 |  |  |
|  | Black/African/Caribbean/Black British – African | 1 | 0.85 |  |  |
|  | Mixed/Multiple ethnic groups – Any other Mixed background | 1 | 0.85 |  |  |
|  | Mixed/Multiple ethnic groups – White and Black African | 1 | 0.85 |  |  |
|  | Other ethnic group – Any other ethnic background | 3 | 2.56 |  |  |
|  | White – Any other White background | 16 | 13.68 |  |  |
|  | White – English/Welsh/Scottish/ Northern Irish/British/Irish | 85 | 72.65 |  |  |
|  | Black/African/Caribbean/Black British – Caribbean | 1 | 0.85 |  |  |
|  | Other ethnic group – Arab | 2 | 1.71 |  |  |
| Height (cm) |  |  |  | 166.37 (7.66) | 149 – 185 |
| Weight (kg) |  |  |  | 96.03 (31.67) | 54.5 – 300 |
| BMI Category | Healthy weight | 18 | 15.38 | 34.76 | 20.02 – |
|  | Overweight | 28 | 23.93 | (11.17) | 106.75 |
|  | Obesity Class I | 24 | 20.51 |  |  |
|  | Obesity Class II | 20 | 17.09 |  |  |
|  | Obesity Class III (Morbid Obesity) | 27 | 23.08 |  |  |

### Table Descriptive Statistics for CRAVED, YFAS, and BES

| Scale | No. of Items | Observed Range | Mean (SD) | Median (IQR) | Cronbach’s α |
| --- | --- | --- | --- | --- | --- |
| CRAVED | 6 | 2 – 6 | 4.99 (1.21) | 5 (2) | 0.576 |
| YFAS | 35 | 0 – 11 | 7.71 (3.22) | 8 (5) | 0.962 |
| BES | 16 | 10 – 44 | 27.06 (8.32) | 27 (13) | 0.891 |

SD: Standard Deviation; IQR: Interquartile Range

### Table CRAVED Item Statistics

| Item | Mean (SD) | Item-Total Correlation | Cronbach’s α if Item Deleted |
| --- | --- | --- | --- |
| CRAVED1 | 0.94 (0.24) | 0.478 (0.000**) | 0.543 |
| CRAVED2 | 0.73 (0.45) | 0.745 (0.000**) | 0.432 |
| CRAVED3 | 0.66 (0.48) | 0.655 (0.000**) | 0.538 |
| CRAVED4 | 0.99 (0.09) | 0.077 (0.412) | 0.604 |
| CRAVED5 | 0.77 (0.42) | 0.654 (0.000**) | 0.502 |
| CRAVED6 | 0.91 (0.29) | 0.558 (0.000**) | 0.519 |

** Highly statistically significant at p ≤ 0.001.
